# Association of oxidative balance score with *helicobacter pylori* infection and mortality in the US population

**DOI:** 10.7150/ijms.95292

**Published:** 2024-05-19

**Authors:** Yujun Xiong, Huazhao Xu, Xingyun Zhu, Zitian Zheng, Qingfeng Luo

**Affiliations:** 1 Department of Gastroenterology, Beijing Hospital, National Center of Gerontology, Institute of Geriatric Medicine, Chinese Academy of Medical Sciences, Beijing, P.R. China. 100370, Beijing, China.; 2 Hospital Administration Office, Beijing Hospital, National Center of Gerontology, Institute of Geriatric Medicine, Chinese Academy of Medical Sciences, Beijing, China.; 3 Department of Endocrinology, Beijing Jishuitan Hospital, No. 31, East Xinjiekou Street, Xicheng District, 100035, Beijing, People's Republic of China.; 4 Department of Orthopedics, Beijing Hospital, National Center of Gerontology, Institute of Geriatric Medicine, Chinese Academy of Medical Sciences, Beijing, PR. China.; 5 Peking University Fifth School of Clinical Medicine, Beijing, PR. China.

**Keywords:** *H. pylori* infection, dietary, lifestyle, OBS, NHANES, mortality

## Abstract

**Background:** Limited research has examined the association between Oxidative Balance Score (OBS) and mortality, particularly in individuals with *Helicobacter pylori (H. pylori)* infection. This study investigates the correlation between OBS and *H. pylori* infection and their impacts on all-cause mortality within a cohort of individuals, considering both infected and uninfected individuals.

**Methods:** Data from the National Health and Nutrition Examination Survey (NHANES) 1999-2018, comprising 4,532 participants, were analyzed. Logistic regression analyses assessed the relationship between *H. pylori* infection and relevant covariates. Cox regression and restricted cubic spline analysis evaluated the association between total OBS, lifestyle OBS, dietary OBS, and all-cause mortality in *H. pylori*-positive and -negative individuals.

**Results:** Restricted cubic spline modeling revealed a linear relationship between total OBS and all-cause mortality, particularly in *H. pylori*-negative patients. Total OBS, dietary OBS, and lifestyle OBS inversely correlated with *H. pylori* infection, even after adjusting for confounders. Higher dietary OBS was associated with decreased mortality risk exclusively in *H. pylori*-positive individuals, while lifestyle OBS was associated with mortality only in *H. pylori*-negative individuals. These findings underscore the complex relationships between OBS, *H. pylori* infection, and mortality, stressing the importance of infection status in assessing oxidative balance's impact on health.

**Conclusion:** In this sample, higher OBS was associated with lower *H. pylori* infection risks. Dietary OBS correlated significantly with all-cause mortality in *H. pylori*-positive individuals, while lifestyle OBS was notably associated with mortality in *H. pylori*-negative participants. Further research is necessary to elucidate the underlying mechanisms and clinical implications of these findings.

## Introduction

*Helicobacter pylori (H. pylori)* infection, a prevalent colonization of the gastric mucosa, infects over 4.4 billion people worldwide, approximately 50% of the world's population 1, and remains a significant etiological factor in various gastrointestinal disorders 2, 3. *H. pylori* infection is closely linked with the development of duodenal ulcers (present in up to 90% of cases) and gastric ulcers (seen in approximately 80% of cases), as well as malignant transformations. *H. pylori* infection is a major risk factor for mucosa-associated lymphoid tissue (MALT) lymphoma and gastric cancer, occurring in as high as 90% of cases 4. Extensive research has been dedicated to unraveling the mechanistic intricacies linking *H. pylori* to oxidative stress, characterized by an imbalance between reactive oxygen species (ROS) production and antioxidative defense mechanisms 5. Activation of inflammatory cascades and perturbations in cellular redox homeostasis are recognized as pivotal contributors to the pathogenesis of *H. pylori*-associated diseases, including gastritis, peptic ulcers, and gastric malignancies 6.

The scope of oxidative stress associated with *H. pylori* infection extends beyond bacterial-induced processes, encompassing lifestyle and dietary factors. Lifestyle choices such as smoking and excessive alcohol consumption contribute to heightened oxidative stress in individuals infected with *H. pylori* 7. Furthermore, existing evidence suggests that specific dietary behaviors, characterized by the consumption of nutritionally suboptimal foods, are correlated with an increased risk of contracting *H. pylori* 8. Furthermore, numerous studies have explored the correlation between oxidative stress and mortality. Within a cohort of Spanish graduates adhering to a Mediterranean diet, there exists a robust inverse association between the Oxidative Balance Score (OBS) and overall mortality, as well as mortality specifically related to cardiovascular disease (CVD) and cancer. The findings suggest a strong link between the OBS and reduced risks of all-cause mortality, CVD-related mortality, and cancer-related mortality within this particular population 9. In a separate investigation utilizing data sourced from the National Health and Nutrition Examination Survey (NHANES) database, findings revealed that higher dietary OBS was associated with lower all-cause mortality 10.

To objectively quantify the impact of diverse dietary components and lifestyles on the comprehensive oxidation/antioxidant system, the concept of the OBS has been introduced. The OBS serves as a metric to assess the overall load of oxidative stress, wherein elevated OBS scores indicated increased exposure to antioxidants, providing a systematic approach to evaluating the interplay between different dietary elements and lifestyle choices on the body's oxidative and antioxidant processes 11, thereby influencing the progression of associated gastrointestinal diseases.

Hence, this study aims to explore the relationship between the OBS and *H. pylori* infection, and investigate whether the associations between OBS, lifestyle OBS, dietary OBS and all-cause mortality are mediated by* H. pylori* infection using the data in the NHANES database.

## Materials and Methods

### Study design and participants

The NHANES initiative employs a refined and intricate methodology to periodically select a representative sample of the U.S. population. Its primary objective involves evaluating the health and nutritional status of individuals in the United States 12. To uphold ethical standards, the survey has garnered approval from The National Center for Health Statistics Institutional Review Board. Furthermore, prior to their inclusion in the study, all participants have willingly provided written informed consent. NHANES encompasses a broad spectrum of data, including demographics, dietary patterns, medical examination results, laboratory findings, and responses to questionnaires 13.

**Figure 1:**
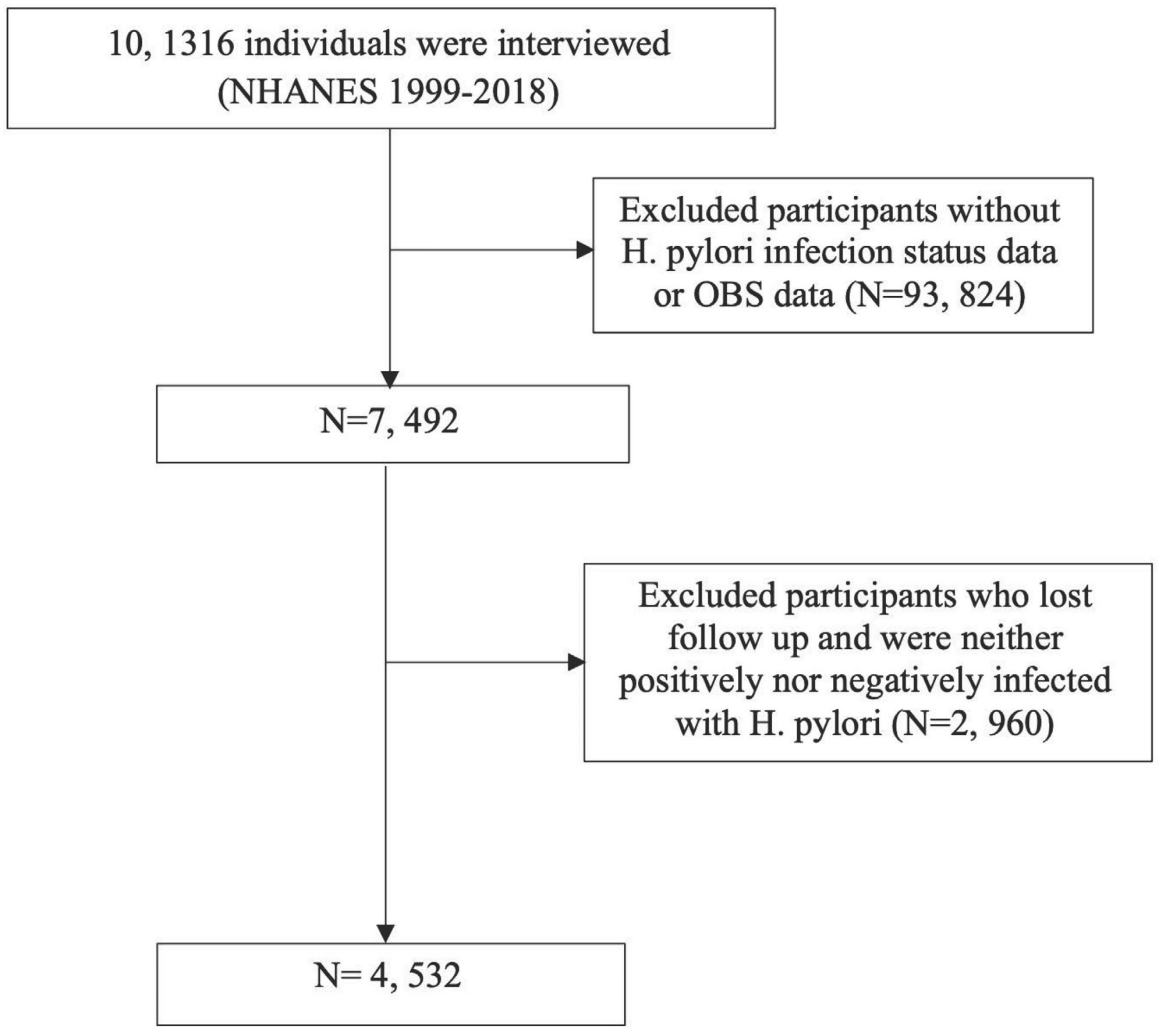
Flow chart for inclusion and exclusion of the study population.

Throughout the NHANES 1999-2018 cycle, the study encompassed 101,136 participants. Following the exclusion of individuals lacking *H. pylori* infection status or OBS data, as well as those who were lost to follow-up or did not meet the criteria for *H. pylori* infection, the remaining subset formed the basis for analysis (Figure 1).

### Oxidative balance score (OBS)

The OBS was calculated by evaluating 16 nutrients and 4 lifestyle factors, encompassing 5 pro-oxidants and 15 antioxidants. This assessment was guided by prior knowledge regarding the association between oxidative stress and these specific nutrients or lifestyle elements 14. Within this study, OBS comprised dietary pro-oxidants (total fat intake and iron intake), dietary antioxidants (β-carotene intake and vitamin C intake, etc.), along with non-dietary lifestyle pro-oxidants (cigarette smoking, alcohol consumption, and BMI), and non-dietary lifestyle antioxidants (physical activity) 15.

### *Helicobacter pylori* status

*H. pylori* immunoglobulin G (IgG) antibodies were identified using an enzyme-linked immunosorbent assay (ELISA) kit produced by Wampole Laboratories (Cranbury, NJ) to quantify the levels of IgG antibodies against *H. pylori* 16. The participants were categorized into two groups: *Hp* positive (optical density (OD) value ≥1.1) and *Hp* negative (OD value <0.9), based on the established ELISA cut-off value. Equivocal results falling within the range of 0.9 to 1.1 were excluded from the analysis to ensure precise statistical outcomes in this study 17.

### Follow up and endpoint

The mortality status and cause of death for participants were ascertained by cross-referencing their records with the publicly accessible National Death Index files, up until December 31, 2019 (https://www.cdc.gov/nchs/data-linkage/mortality.htm). The median follow-up time for *H. pylori*-positive individuals was 235 months (interquartile range 184, 243), while for *H. pylori*-negative patients, it stood at 236 months (interquartile range 229, 242).

### Covariate

Several clinical data, recognized as covariates, were included because of their potential influence on the relationship between OBS and *H. pylori* infection, including age, sex, race, poverty, education, smoking habits, alcohol drinking, diabetes mellitus, hypertension status, cardiovascular disease, blood lipids and glucose, lifestyle OBS, dietary OBS and OBS. Hypertension was defined as self-reported hypertension, a systolic blood pressure (SBP) of ≥ 140 mmHg, a diastolic blood pressure (DBP) of ≥ 90 mmHg, or the use of antihypertensive medications 18. Smoking status was classified into three groups: former, never and now. Former smokers were defined as those who had previously smoked at least 100 cigarettes but were not presently smoking. Never-smokers included individuals who had either never smoked or had smoked fewer than 100 cigarettes in their lifetime. Now-smokers were participants who had smoked at least 100 cigarettes in their lifetime and reported consuming a non-zero number of cigarettes per day within the past 30 days 8. Alcohol drinking status was classified into four distinct categories, reflecting their alcohol consumption patterns: Never drinkers (lifetime abstainers), former drinkers (abstinent within the past year), moderate drinkers (1 or 2 drinks per day for females/males, respectively), and heavy drinkers (>1 or >2 drinks per day for females/males, respectively, and/or frequent binge drinking) 11, 19. Moreover, the educational level was categorized into three groups: less than high school, high school, and more than high school.

### Statistical analysis

The baseline characteristics of participants were summarized and compared between *H. pylori*-infected and uninfected patients. Continuous variables were expressed as mean (±SD) and compared using either a t-test or Wilcoxon rank-sum test, based on the outcome of the Kolmogorov-Smirnov normality test. Categorical variables were presented as frequency (percentage) and compared using the Chi-square test.

Both univariable and multivariable-adjusted logistic regression analyses were applied to determine the odds ratio (OR) alongside a 95% confidence interval (CI) for assessing the relationship between OBS and *H. pylori* infection. Additionally, the potential nonlinear connections between OBS and all-cause mortality were explored using restricted cubic spline (RCS) curves. These curves were positioned at specific percentiles (5%, 35%, 65%, and 95%) within the OBS distribution 20. Examining the association of OBS with all-cause mortality, cox proportional hazards models were utilized to compute hazard ratios (HRs) and their corresponding 95% CIs. To avoid overadjustment and optimize data utilization for variables related to OBS, lifestyle OBS, dietary OBS, and mortality, three models were developed: model 1 adjusted for age, sex, and BMI; model 2 included adjustments for age, sex, BMI, race, education, smoking, and alcohol consumption; and model 3 encompassed fasting total cholesterol (TC), diabetes mellitus, and hypertension in addition to the adjustments in model 2. Categorizing OBS as a continuous variable into tertiles, cox proportional hazards models were implemented, using tertile 1 (T1 group) as the reference. The event-free survival rates among these tertile groups were estimated using the Kaplan-Meier method and compared through the log-rank test.

A two-sided *P*< 0.05 was considered statistically significant. All analyses were performed using SPSS version 26.0 (IBM Corp, Armonk, NY) and R (version 4.3.2) 21, 22.

## Results

### Study participants and baseline characteristics

In the final cohort, 4,532 American adults were included, among whom 1,970 participants tested positive for *H. pylori* (**Figure 1** and **Table 1**). The average age was 46.28±20.21 years, with males accounting for 47.0% of the sample. The mean OBS was 17.59±7.71, with the lifestyle OBS at 3.30±1.45 and the dietary OBS mirroring the mean OBS at 17.59±7.71. More specifically, within the H. pylori positive group, there was a greater proportion of older individuals, males, individuals with lower socioeconomic status and educational attainment, current smokers, former drinkers, individuals with diabetes, hypertension, higher body mass index (BMI), and elevated blood lipid levels. Moreover, this group showed a notably higher prevalence of atherosclerotic cardiovascular disease (ASCVD) events and associated conditions.

### Associations between OBS and *H. pylori* infection

We conducted linear regression analysis to investigate the relationship between variables and *H. pylori* infection in adults, as outlined in Table 2. In the multivariate analysis, demographic factors such as age, sex, BMI, race, and education all exhibited significant associations with *H. pylori* infection. Alcohol consumption demonstrated an association with *H. pylori* status (*P* = 0.047), whereas smoking did not show a significant association with *H. pylori* (*P* = 0.594). Notably, OBS, lifestyle OBS, and dietary OBS all displayed statistically significant associations with *H. pylori* status.

Subsequently, a multivariable logistic regression analysis was conducted to investigate the association between total/dietary/lifestyle OBS and the risk of *H. pylori* infection, as delineated in Table 3. When total OBS levels were examined as a continuous variable in model 3, a one standard deviation (SD) increase in OBS resulted in an adjusted odds ratio (OR) for *H. pylori* infection of 0.982 (95% CI: 0.975-0.989). Assessing total OBS as tertiles in the initial model (model 0), participants in the two higher tertiles of OBS (T2, T3) demonstrated a significantly lower risk of *H. pylori* infection compared to those in the lowest tertile (T1). This negative correlation between *H. pylori* infection and OBS persisted in both T2 and T3 groups. This pattern continued after adjusting for age, sex, and BMI in model 1. Notably, the risk of *H. pylori* infection remained significantly lower in the T3 tertile compared to T1, with an OR of 0.601 and a 95% CI of (0.534,0.675). Further adjustments for races, education, smoking, and alcohol consumption in model 2 and additional adjustments for comorbidities, such as fasting total cholesterol, diabetes mellitus, and hypertension in model 3, individuals in the highest OBS tertile had the lowest risk of *H. pylori* infection compared to those in the lowest tertile, revealing the negative correlation between *H. pylori* infection and OBS. It was observed that both lifestyle and dietary OBS were negatively correlated with *H. pylori* infection as continuous variable and categorized variables, even after adjustment for several risk factors.

### Correlation between OBS and all-cause mortality

**Figure 2:**
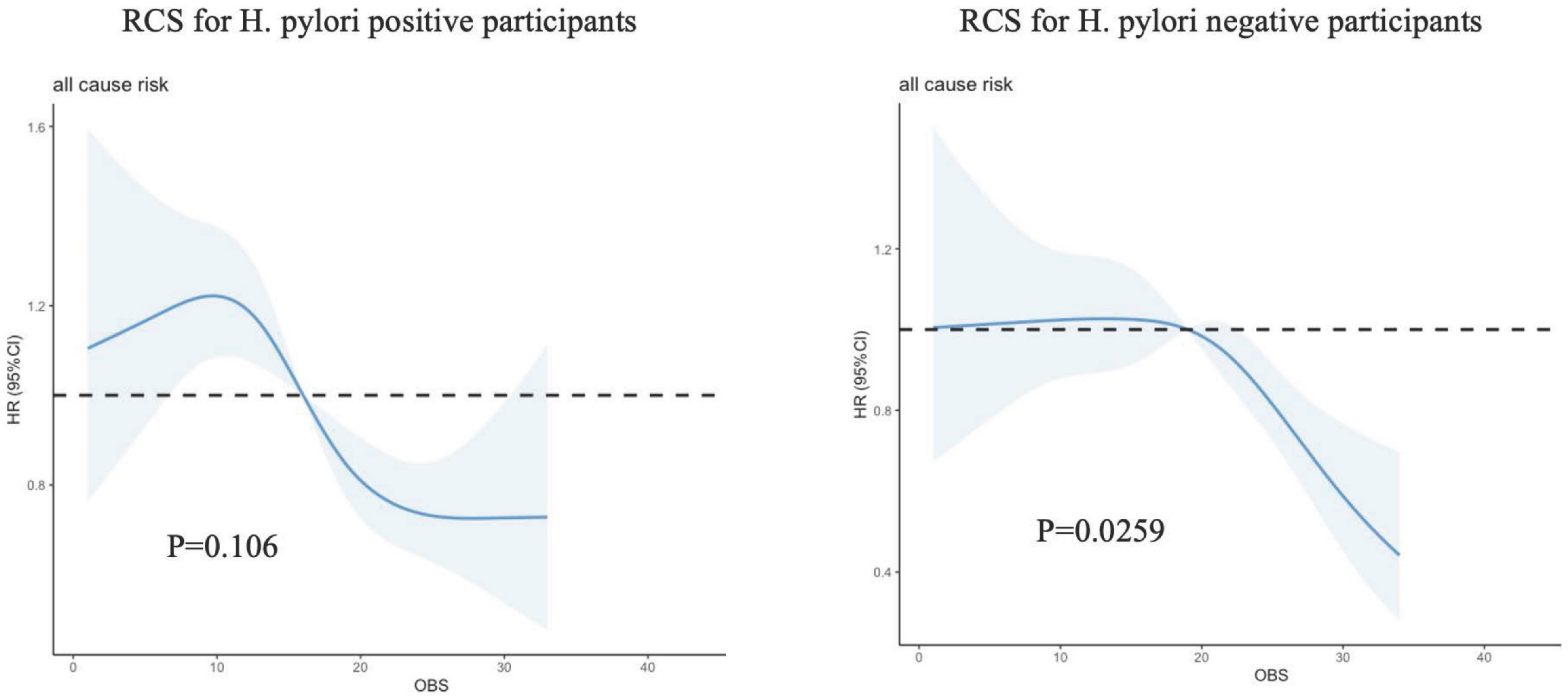
Restricted cubic spline (RCS) for the association between OBS and the risks of all-cause death in patients with or without *H. pylori* infection.

Among the total participants, a total of 1,296 individuals (28.60%) experienced death. The relationship between total OBS and all-cause mortality was additionally examined through restricted cubic spline (RCS) curves, as illustrated in Figure 2. The RCS analysis demonstrated that total OBS, as a continuous variable, was linked to a reduced adjusted risk of all-cause mortality in *H. pylori* uninfected patients (*P*=0.0259).

**Figure 3:**
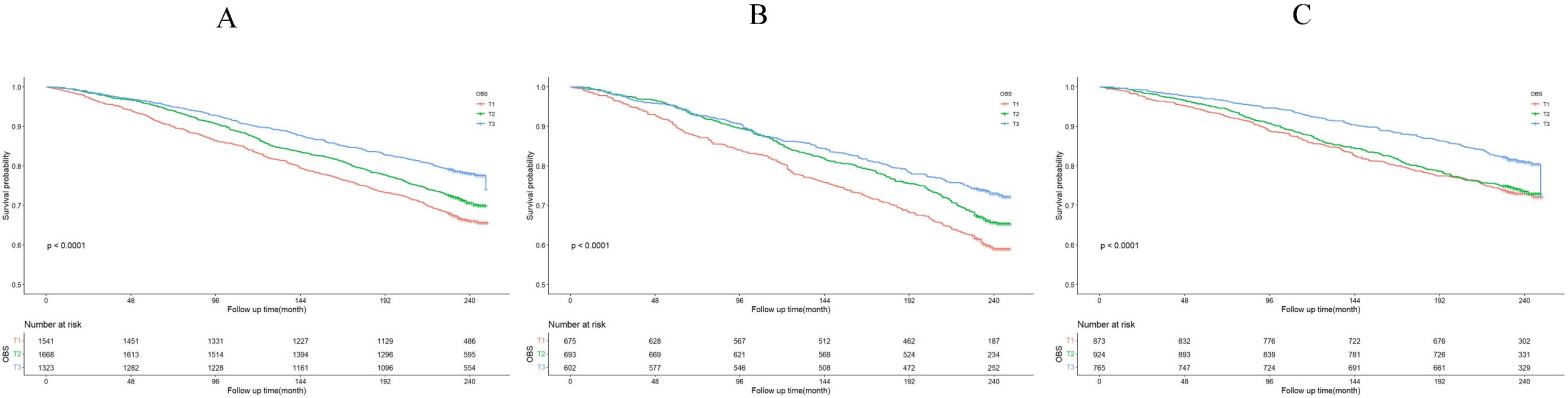
OBS association with all-cause mortality. (A) Kaplan-Meier survival estimates for all-cause mortality in all participants; (B) Kaplan-Meier survival estimates for all-cause mortality in *H. pylori* positively infected participants; (C) Kaplan-Meier survival estimates for all-cause mortality in *H. pylori* negatively infected participants.

**Figure 4:**
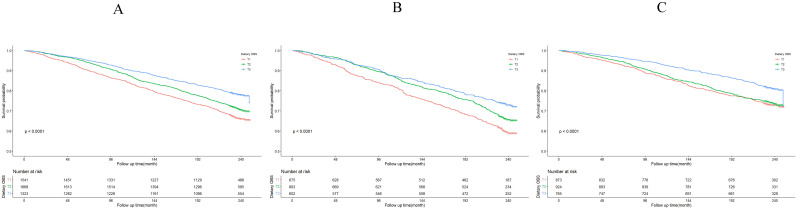
Lifestyle OBS association with all-cause mortality. (A) Kaplan-Meier survival estimates for all-cause mortality in all participants; (B) Kaplan-Meier survival estimates for all-cause mortality in *H. pylori* positively infected participants; (C) Kaplan-Meier survival estimates for all-cause mortality in *H. pylori* negatively infected participants.

**Figure 5:**
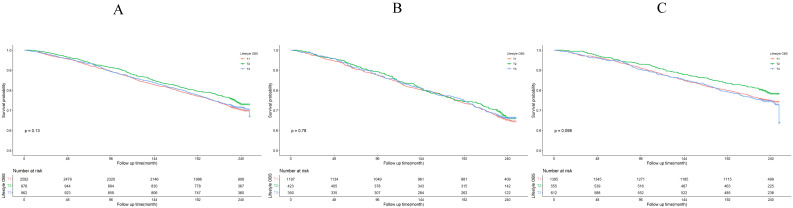
Dietary OBS association with all-cause mortality. (A) Kaplan-Meier survival estimates for all-cause mortality in all participants; (B) Kaplan-Meier survival estimates for all-cause mortality in *H. pylori* positively infected participants; (C) Kaplan-Meier survival estimates for all-cause mortality in *H. pylori* negatively infected participants.

The Kaplan-Meier survival curve analysis demonstrated that higher total OBS were associated with lower all-cause mortality (depicted in Figure 3). Notably, higher lifestyle OBS was not associated with lower all-cause mortality in *H. pylori* negatively infected participants (*P*-log rank = 0.098, as shown in Fig. 4C). Conversely, in *H. pylori* positively infected participants, both lifestyle and dietary OBS were negatively associated with mortality (depicted in Fig. 5B and 5C). As a continuous variable, in model 3, for every one standard deviation (SD) increase in total OBS, the adjusted hazard ratio (HR) for mortality was 0.974 (95% CI: 0.962-0.986) in *H. pylori*-positive participants and 0.992 (95% CI: 0.979-1.004) in *H. pylori*-negative population. When specified as lifestyle and dietary subgroups of total OBS, after adjusting for potential risk factors, including age, sex, BMI, race, education, smoking, alcohol consumption, fasting total cholesterol, diabetes mellitus, and hypertension, the T3 group of dietary OBS (HR: 0.608, 95% CI: 0.490-0.756; *P* < 0.001) exhibited a lower risk of death in *H. pylori* positively infected individuals (Table 4), while in *H. pylori* negatively infected participants, the T3 group of the lifestyle OBS (HR: 0.737, 95% CI: 0.581-0.934; *P* = 0.012) displayed a similar phenomenon (Table 5).

## Discussion

Our study explored the correlation between the OBS and *H. pylori* infection, as well as the overall mortality in cohorts with and without *H. pylori* infection, using data spanning NHANES 1999-2018. Our findings revealed that individuals exhibiting higher total OBS, dietary OBS, and lifestyle OBS experienced diminished risks of *H. pylori* infection. Notably, the impact of anti-inflammatory diet, as indicated by an elevated dietary OBS, on mortality was exclusively evident among those who tested positive for *H. pylori* infection. Conversely, lifestyle OBS demonstrated a negative association with mortality in participants without *H. pylori* infection.

Current evidence indicates a pivotal role of oxidative stress in the onset of *H. pylori* infection, underscoring the importance of identifying antioxidant elements with protective effects from a public health standpoint 23. In a Mediterranean cohort of Spanish graduates, a robust inverse association was identified between the OBS and mortality rates related to all causes, cardiovascular diseases (CVD), and cancer 24. ROS and reactive nitrogen species generated by both immune and epithelial cells inflict damage upon host cells, potentially leading to DNA damage. *H. pylori* has evolved mechanisms to trigger this detrimental response while simultaneously attenuating the host's defenses aimed at eliminating the bacteria. This sustained condition of inflammation and oxidative stress has the potential to contribute to gastric carcinogenesis, which therefore cause final death 25.

Significant differences were observed in the correlations between dietary OBS and *H. pylori* infection, partially aligning with findings from previous research 26. In an Iranian case-control study, it was observed that a suitable intake of nutrient antioxidants might contribute to reducing the probability of *H. pylori* infection risk 27. Remarkably, several studies have recorded a significant correlation between the dietary OBS and diverse inflammation-related conditions, encompassing non-alcoholic fatty liver disease 28, chronic kidney disease 29, and chronic lung disease 14. These findings highlight the significance of evaluating the overall diet to comprehend the relationship between dietary factors and disease outcomes. Consistent with previous research that has shown a persistent inverse association between the diet antioxidant index (DAI) and *H. pylori* infection 27, our study also demonstrated that dietary OBS was inversely associated with *H. pylori* infection and, consequently, mortality.

For *H. pylori*-negative patients, our study reveals that lifestyle factors, encompassing BMI, smoking, alcohol consumption, and physical activity, intricately influence mortality outcomes. In another longitudinal cohort study, individuals with *H. pylori* infection, lacking heavy drinking or smoking habits, displayed a noteworthy association with gastric neoplasia. Conversely, non-*H. pylori* drinkers showed no discernible association, while non-*H. pylori* smokers exhibited a significant correlation with gastric neoplasia. These findings underscore the intricate relationship between *H. pylori* infection, lifestyle factors, and the development of gastric neoplasia over time, emphasizing the need for a comprehensive understanding of both infectious and behavioral contributors to gastric health outcomes 30. The mechanisms underlying this association are multifaceted. Abstaining from smoking and moderate alcohol intake in this context demonstrates a protective effect, potentially mitigating inflammation and oxidative stress 31, 32.

To the best of our knowledge, our study represents the initial investigation into the association between the OBS and *H. pylori* infection, offering valuable insights to augment the existing body of knowledge.

## Limitations

Firstly, a single 24-hour dietary recall method was employed to evaluate dietary pro-oxidants and antioxidants, which may not fully capture day-to-day variability in dietary patterns. Despite the potential limitation associated with this approach, it offers advantages such as reduced susceptibility to recall bias. Secondly, the significant association between OBS and *H. pylori* infection could be influenced by unmeasured or residual confounding factors 33.

## Conclusion

In our sample, higher OBS was linked to lower *H. pylori* infection risks. Dietary OBS correlated significantly with all-cause mortality in *H. pylori*-positive individuals, while lifestyle OBS was notably associated with mortality in *H. pylori*-negative participants. These outcomes emphasized OBS's independent predictive value, providing valuable insights into the dietary and lifestyle choices of those with *H. pylori* infection.

## Figures and Tables

**Table 1:** Baseline characteristics of participants with different *H. pylori* infection status.

	Overall (n=4532)	*Hp* negative (n=2562)	*Hp* positive (n=1970)	*P* value
Age (years)	46.28 (20.21)	43.45 (20.12)	49.95 (19.73)	<0.001
Sex (male %)	2130 (47.0)	1166 (45.5)	964 (48.9)	0.025
BMI (kg/m^2^)	28.06 (6.25)	27.86 (6.29)	28.33 (6.19)	0.011
Race (%)				<0.001
Mexican American	1332 (29.4)	488 (19.0)	844 (42.8)	
Non-Hispanic Black	842 (18.6)	381 (14.9)	461 (23.4)	
Non-Hispanic White	1931 (42.6)	1486 (58.0)	445 (22.6)	
Other Hispanic	280 (6.2)	118 (4.6)	162 (8.2)	
Other Race	148 (3.3)	89 (3.5)	59 (3.0)	
Poverty income ratio	2.46 (1.61)	2.78 (1.64)	2.03 (1.46)	<0.001
Education (%)				<0.001
High School	1060 (23.5)	668 (26.1)	392 (20.0)	
Less Than High School	1784 (39.5)	667 (26.1)	1117 (57.0)	
More Than High School	1676 (37.1)	1224 (47.8)	452 (23.0)	
Fasting insulin (uU/mL)	13.97 (13.16)	13.29 (10.67)	14.87 (15.83)	0.006
Fasting glucose(mg/dL)	102.55 (35.45)	98.84 (27.23)	107.47 (43.60)	<0.001
HbA1c (%)	5.51 (1.11)	5.36 (0.91)	5.70 (1.29)	<0.001
Total bilirubin (umol/L)	9.69 (5.02)	9.76 (5.33)	9.61 (4.58)	0.307
Creatinine(mg/dL)	0.74 (0.55)	0.74 (0.56)	0.74 (0.55)	0.822
Fasting total cholesterol (mg/dL)	201.18 (42.52)	200.04 (42.05)	202.66 (43.11)	0.04
Fasting triglyceride(mg/dL)	144.35 (107.41)	135.94 (91.76)	155.52 (124.40)	<0.001
LDL (mg/dL)	123.05 (35.46)	122.29 (35.35)	124.12 (35.62)	0.26
HDL (mg/dL)	51.04 (15.22)	52.07 (15.38)	49.70 (14.89)	<0.001
ASCVD = yes (%)	381 (9.4)	183 (8.2)	198 (11.0)	0.003
Smoke (%)				0.048
Former	1074 (26.7)	588 (26.4)	486 (27.0)	
Never	2130 (52.9)	1213 (54.4)	917 (51.0)	
Now	821 (20.4)	427 (19.2)	394 (21.9)	
Alcohol drink (%)				<0.001
Former	781 (20.4)	371 (17.3)	410 (24.4)	
Heavy	726 (19.0)	396 (18.5)	330 (19.6)	
Mild	1221 (32.0)	745 (34.8)	476 (28.3)	
Moderate	518 (13.6)	324 (15.1)	194 (11.5)	
Never	575 (15.0)	303 (14.2)	272 (16.2)	
Hypertension (%)	1672 (36.9)	839 (32.7)	833 (42.3)	<0.001
SBP (mmHg)	125.23 (20.99)	122.63 (19.51)	128.63 (22.33)	<0.001
DBP (mmHg)	70.42 (14.33)	70.04 (13.84)	70.93 (14.95)	0.041
Diabetes Mellitus (%)	518 (12.1)	214 (9.0)	304 (16.1)	<0.001
CRP (mg/dL)	0.50 (0.98)	0.47 (0.98)	0.53 (0.97)	0.033
OBS dietary	17.59 (7.71)	18.53 (7.81)	16.37 (7.42)	<0.001
OBS lifestyle	3.30 (1.45)	3.39 (1.47)	3.19 (1.41)	<0.001
OBS	17.59 (7.71)	18.53 (7.81)	16.37 (7.42)	<0.001

Abbreviation: Helicobacter Pylori (Hp), systolic blood pressure (SBP), diastolic blood pressure (DBP), body mass index (BMI), glycated hemoglobin (HbA1c), high-density lipoprotein (HDL), low-density lipoprotein (LDL), C-reactive protein (CRP), atherosclerotic cardiovascular disease (ASCVD), oxidative balance score (OBS).

**Table 2:** Risk factors for *H. pylori* infection in adults in NHANES 1999-2018

Variables	β	Standard Error	95% CI	*P* value
Age	0.01	0.00	0.01 ~ 0.01	<0.001
Sex	0.03	0.01	0.01 ~ 0.06	0.022
Race	-0.36	0.01	-0.38 ~ -0.33	<0.001
Education	0.08	0.02	0.05 ~ 0.12	<0.001
BMI	0.01	0.00	0.01 ~ 0.01	0.011
Poverty	-0.07	0.00	-0.08 ~ -0.06	<0.001
Diabetes mellitus	-0.17	0.02	-0.22 ~ -0.13	<0.001
Fasting total cholesterol	0.01	0.00	0.01 ~ 0.01	0.042
ASCVD	0.08	0.03	0.03 ~ 0.13	0.002
Hypertension	0.10	0.02	0.07 ~ 0.13	<0.001
Smoke	0.01	0.01	-0.02 ~ 0.04	0.594
Alcohol drink	-0.04	0.02	-0.09 ~ -0.01	0.047
Dietary OBS	-0.01	0.00	-0.01 ~ -0.01	<0.001
lifestyle OBS	-0.02	0.01	-0.03 ~ -0.01	<0.001
OBS	-0.01	0.00	-0.01 ~ -0.01	<0.001

**Table 3:** Associations between the dietary/lifestyle OBS and *H. pylori* infection.

Variables	OR (95%CI)			
OBS	Model 0 0.971(0.966,0.977) ***	Model 1^a^ 0.970(0.964,0.976) ***	Model 2^b^ 0.984(0.977,0.991) ***	Model 3^c^ 0.982(0.975,0.989) ***
T1 group	Ref	Ref	Ref	Ref
T2 group	0.831(0.751,0.919) ***	0.816(0.738,0.904) ***	0.912(0.816,1.020)	0.906(0.808,1.014)
T3 group	0.593(0.528,0.666) ***	0.601(0.534,0.675) ***	0.793(0.696,0.904) ***	0.770(0.672,0.881) ***
Lifestyle OBS	0.939(0.910,0.968) ***	0.894(0.863,0.927) ***	0.956(0.916,0.998) *	0.952(0.911,0.995) *
T1 group	Ref	Ref	Ref	Ref
T2 group	0.891(0.797,0.995) *	0.837(0.747,0.939) **	0.913(0.802,1.040)	0.908(0.795,1.038)
T3 group	0.762(0.677,0.859) ***	0.685(0.603,0.779) ***	0.839(0.725,0.971) *	0.814(0.700,0.946) **
Dietary OBS	0.971(0.966,0.977) ***	0.970(0.964,0.976) ***	0.984(0.977,0.991) ***	0.982(0.975,0.989) ***
T1 group	Ref	Ref	Ref	Ref
T2 group	0.831(0.751,0.919) ***	0.816(0.738,0.904) ***	0.912(0.816,1.020)	0.906(0.808,1.014)
T3 group	0.593(0.528,0.666) ***	0.601(0.534,0.675) ***	0.793(0.696,0.904) ***	0.770(0.672,0.881) ***

^a^ Model 1 adjusted for age, sex, BMI ^b^ Model 2 adjusted for age, sex, BMI, races, education, smoke and alcohol drink ^c^ Model 3 adjusted for age, sex, BMI, races, education, smoke, alcohol drink, fasting total cholesterol, diabetes mellitus and hypertension. *P < 0.05, **P < 0.01, ***P < 0.001.

**Table 4:** Associations of the dietary/lifestyle OBS and all-cause mortality in *H. pylori* positively infected participants.

Variables	HR (95%CI)			
OBS	Model 0 0.975(0.965,0.985) ***	Model 1^a^ 0.972(0.961,0.983) ***	Model 2^b^ 0.979(0.967,0.991) ***	Model 3^c^ 0.974(0.962,0.986) ***
T1 group	Ref	Ref	Ref	Ref
T2 group	0.789(0.662,0.939) **	0.700(0.586,0.836) ***	0.756(0.625,0.913) **	0.696(0.574,0.844) ***
T3 group	0.610(0.502,0.741) ***	0.577(0.474,0.703) ***	0.649(0.524,0.803) ***	0.608(0.490,0.756) ***
Lifestyle OBS	1.001(0.949,1.057)	0.848(0.796,0.903) ***	0.939(0.873,1.010)	0.949(0.882,1.022)
T1 group	Ref	Ref	Ref	Ref
T2 group	0.938(0.774,1.136)	0.684(0.560,0.837) ***	0.828(0.664,1.031)	0.840(0.672,1.050)
T3 group	0.961(0.782,1.180)	0.607(0.486,0.758) ***	0.768(0.603,0.978) *	0.809(0.634,1.031)
Dietary OBS	0.975(0.965,0.985) ***	0.972(0.961,0.983) ***	0.979(0.967,0.991) ***	0.974(0.962,0.986) ***
T1 group	Ref	Ref	Ref	Ref
T2 group	0.789(0.662,0.939) **	0.700(0.586,0.836) ***	0.756(0.625,0.913) **	0.696(0.574,0.844) ***
T3 group	0.610(0.502,0.741) ***	0.577(0.474,0.703) ***	0.649(0.524,0.803) ***	0.608(0.490,0.756) ***

^a^ Model 1 adjusted for age, sex, BMI ^b^ Model 2 adjusted for age, sex, BMI, races, education, smoke and alcohol drink ^c^ Model 3 adjusted for age, sex, BMI, races, education, smoke, alcohol drink, fasting total cholesterol, diabetes mellitus and hypertension. *P < 0.05, **P < 0.01, ***P < 0.001.

**Table 5:** Associations of the dietary/lifestyle OBS and all-cause mortality in *H. pylori* negatively infected participants.

Variables	HR (95%CI)			
OBS	Model 0 0.980(0.971,0.990) ***	Model 1^a^ 0.977(0.967,0.988) ***	Model 2^b^ 0.990(0.978,1.002)	Model 3^c^ 0.992(0.979,1.004)
T1 group	Ref	Ref	Ref	Ref
T2 group	0.955(0.799,1.142)	0.916(0.764,1.098)	1.036(0.855,1.255)	1.081(0.890,1.313)
T3 group	0.654(0.532,0.804) ***	0.673(0.545,0.830) ***	0.849(0.673,1.071)	0.863(0.683,1.092)
Lifestyle OBS	1.003(0.951,1.058)	0.856(0.805,0.910) ***	0.914(0.853,0.979) *	0.927(0.863,0.995) *
T1 group	Ref	Ref	Ref	Ref
T2 group	0.814(0.660,1.003)	0.705(0.566,0.879) **	0.818(0.648,1.032)	0.822(0.649,1.039)
T3 group	1.036(0.859,1.250)	0.597(0.481,0.742) ***	0.713(0.564,0.902) **	0.737(0.581,0.934) *
Dietary OBS	0.980(0.971,0.990) ***	0.977(0.967,0.988) ***	0.990(0.978,1.002)	0.992(0.979,1.004)
T1 group	Ref	Ref	Ref	Ref
T2 group	0.955(0.799,1.142)	0.916(0.764,1.098)	1.036(0.855,1.255)	1.081(0.890,1.313)
T3 group	0.654(0.532,0.804) ***	0.673(0.545,0.830) ***	0.849(0.673,1.071)	0.863(0.683,1.092)

^a^ Model 1 adjusted for age, sex, BMI ^b^ Model 2 adjusted for age, sex, BMI, races, education, smoke and alcohol drink ^c^ Model 3 adjusted for age, sex, BMI, races, education, smoke, alcohol drink, fasting total cholesterol, diabetes mellitus and hypertension. *P < 0.05, **P < 0.01, ***P < 0.001.

## References

[1] Ali A, AlHussaini KI (2024). Helicobacter pylori: A Contemporary Perspective on Pathogenesis, Diagnosis and Treatment Strategies. Microorganisms.

[2] Addissouky TA, Wang Y, El Sayed IET, Baz AE, Ali MMA, Khalil AA (2023). Recent trends in Helicobacter pylori management: harnessing the power of AI and other advanced approaches. Beni-Suef University Journal of Basic and Applied Sciences.

[3] Hooi JKY, Lai WY, Ng WK, Suen MMY, Underwood FE, Tanyingoh D (2017). Global Prevalence of Helicobacter pylori Infection: Systematic Review and Meta-Analysis. Gastroenterology.

[4] Shatila M, Thomas AS (2022). Current and Future Perspectives in the Diagnosis and Management of Helicobacter pylori Infection. J Clin Med.

[5] Sah DK, Arjunan A, Lee B, Jung YD (2023). Reactive Oxygen Species and H. pylori Infection: A Comprehensive Review of Their Roles in Gastric Cancer Development. Antioxidants (Basel).

[6] Liu Y, Shi Y, Han R, Liu C, Qin X, Li P (2023). Signaling pathways of oxidative stress response: the potential therapeutic targets in gastric cancer. Front Immunol.

[7] Malfertheiner P, Camargo MC, El-Omar E, Liou J-M, Peek R, Schulz C (2023). Helicobacter pylori infection. Nature Reviews Disease Primers.

[8] Sreeja SR, Le TD, Eom BW, Oh SH, Shivappa N, Hebert JR (2022). Association between the Dietary Inflammatory Index and Gastric Disease Risk: Findings from a Korean Population-Based Cohort Study. Nutrients.

[9] Talavera-Rodriguez I, Fernandez-Lazaro CI, Hernández-Ruiz Á, Hershey MS, Galarregui C, Sotos-Prieto M (2023). Association between an oxidative balance score and mortality: a prospective analysis in the SUN cohort. European Journal of Nutrition.

[10] Liu J, Wang W, Wen Y (2023). Association of dietary oxidative balance score and sleep duration with the risk of mortality: prospective study in a representative US population. Public Health Nutr.

[11] Zhang W, Peng SF, Chen L, Chen HM, Cheng XE, Tang YH (2022). Association between the Oxidative Balance Score and Telomere Length from the National Health and Nutrition Examination Survey 1999-2002. Oxid Med Cell Longev.

[12] Dwyer J, Picciano MF, Raiten DJ, Members of the Steering C, National H, Nutrition Examination S (2003). Collection of food and dietary supplement intake data: What We Eat in America-NHANES. J Nutr.

[13] N.C.f.H.S US CENTERS FOR DISEASE CONTROL AND PREVENTION, NATIONAL HEALTH AND NUTRITION EXAMINATION SURVEY 1999-2000, 2001-2002, 2003-2004, 2005-2006, 2011-2012, 2013-2014, 2015-2016, 2017-2018 Documentation Files. 2021.

[14] Xu Z, Xue Y, Wen H, Chen C (2022). Association of oxidative balance score and lung health from the National Health and Nutrition Examination Survey 2007-2012. Front Nutr.

[15] Liu X, Liu X, Wang Y, Zeng B, Zhu B, Dai F (2023). Association between depression and oxidative balance score: National Health and Nutrition Examination Survey (NHANES) 2005-2018. J Affect Disord.

[16] Berrett AN, Gale SD, Erickson LD, Brown BL, Hedges DW (2016). Folate and Inflammatory Markers Moderate the Association Between Helicobacter pylori Exposure and Cognitive Function in US Adults. Helicobacter.

[17] Huang J, Liu Z, Ma J, Liu J, Lv M, Wang F (2022). The Association between Helicobacter pylori Seropositivity and Bone Mineral Density in Adults. Mediators Inflamm.

[18] Wu M, Si J, Liu Y, Kang L, Xu B (2023). Association between composite dietary antioxidant index and hypertension: insights from NHANES. Clin Exp Hypertens.

[19] Warner JB, Zirnheld KH, Hu H, Floyd A, Kong M, McClain CJ (2022). Analysis of alcohol use, consumption of micronutrient and macronutrients, and liver health in the 2017-2018 National Health and Nutrition Examination Survey. Alcohol Clin Exp Res.

[20] Ma W, Yan Z, Wu W, Li D, Zheng S, Lyu J (2021). Dose-Response Association of Waist-to-Height Ratio Plus BMI and Risk of Depression: Evidence from the NHANES 05-16. Int J Gen Med.

[21] Wallace DA, Johnson DA, Redline S, Sofer T, Kossowsky J (2023). Rest-activity rhythms across the lifespan: cross-sectional findings from the US representative National Health and Nutrition Examination Survey. Sleep.

[22] Lei X, Xu Z, Chen W (2023). Association of oxidative balance score with sleep quality: NHANES 2007-2014. J Affect Disord.

[23] Tsukanov VV, Smirnova OV, Kasparov EV, Sinyakov AA, Vasyutin AV, Tonkikh JL (2022). Dynamics of Oxidative Stress in Helicobacter pylori-Positive Patients with Atrophic Body Gastritis and Various Stages of Gastric Cancer. Diagnostics (Basel).

[24] Talavera-Rodriguez I, Fernandez-Lazaro CI, Hernández-Ruiz Á, Hershey MS, Galarregui C, Sotos-Prieto M (2023). Association between an oxidative balance score and mortality: a prospective analysis in the SUN cohort. Eur J Nutr.

[25] Butcher LD, den Hartog G, Ernst PB, Crowe SE (2017). Oxidative Stress Resulting From Helicobacter pylori Infection Contributes to Gastric Carcinogenesis. Cell Mol Gastroenterol Hepatol.

[26] Xiong Y-J, Du L-L, Diao Y-L, Wen J, Meng X-B, Gao J (2023). Association of dietary inflammatory index with helicobacter pylori infection and mortality among US population. Journal of Translational Medicine.

[27] Ebrahimi Z, Masoodi M, Aslani Z, Naghshi S, Khalighi Sikaroudi M, Shidfar F (2022). Association between dietary antioxidant index and risk of Helicobacter pylori infection among adults: a case-control study. BMC Gastroenterol.

[28] Cho AR, Kwon YJ, Lee JH (2023). Oxidative balance score is inversely associated with the incidence of non-alcoholic fatty liver disease. Clin Nutr.

[29] Son DH, Lee HS, Seol SY, Lee YJ, Lee JH (2023). Association between the Oxidative Balance Score and Incident Chronic Kidney Disease in Adults. Antioxidants (Basel).

[30] Usui G, Matsusaka K, Huang KK, Zhu F, Shinozaki T, Fukuyo M Integrated environmental, lifestyle, and epigenetic risk prediction of primary gastric neoplasia using the longitudinally monitored cohorts. EBioMedicine.

[31] Lombardo M, Feraco A, Camajani E, Caprio M, Armani A (2023). Health Effects of Red Wine Consumption: A Narrative Review of an Issue That Still Deserves Debate. Nutrients.

[32] Taylor GMJ, Treur JL (2023). An application of the stress-diathesis model: A review about the association between smoking tobacco, smoking cessation, and mental health. Int J Clin Health Psychol.

[33] Yuan Y, Tan W, Huang Y, Huang H, Li Y, Gou Y (2023). Association between oxidative balance score and urinary incontinence in females: results from the national health and nutrition examination survey in 2005-2018. Int Urol Nephrol.

